# Book Reviews

**DOI:** 10.1038/sj.bjc.6690671

**Published:** 1999-09

**Authors:** Helena Earl

**Affiliations:** Oncology Centre University of Cambridge, Box 193 Addenbrooke's Hospital Hills Road, Cambridge, CB2 2QQ


British Journal of Cancer (1999) 81(1), 187–188
© 1999 Cancer Research Campaign
Article no. bjoc.1999.0671

### Book Reviews

### Cancer Chemotherapy Treatment Protocols

Khayat, Waxman and Antoine, Publication details: Price £24.95, ISBN 0–86542–693–7. Blackwell Science; UK, 1998.

The authors of this text should be applauded for aiming to provide a comprehensive chemotherapy protocol book and easy reference text for the practicing oncologist. Do they achieve their goal? They have certainly provided a broad compilation of the majority of commonly used chemotherapy protocols for malignant disease, which will serve as a good reference text in oncology departments.

The book has an interesting and unusual style and format. There is a short introductory section entitled ‘General Principles’, which covers supportive therapy, and toxicity and administration guidelines. I found this section suffered from being too brief. The authors should have been able to cover toxicity and administration for all the commonly used cytotoxic drugs in some detail. In the supportive therapy section, there are some idiosyncrasies of treatment with which I would not necessarily agree. Unfortunately, because the book is not referenced in the conventional way, it is not possible to access the source easily and examine the evidence for the guidelines. For example, in the treatment of extravasation of vesicant cytotoxic drugs, the authors suggest using 99% dimethyl sulphoxide four times daily for 2 weeks and reference a randomized study in rats. These recommendations would certainly not be current standard practice in oncology centres, but if it is true, we need to see the evidence for it so that improvements in practice can be introduced. For the commonly used drugs some important information is emphasized, which will guide good clinical practice, but in my view, this section should have been more comprehensive.

The main section of the book itemises the majority of protocols used over the past 25 years. In the larger chapters (e.g. on breast cancer) it is difficult to discover how the protocols have been ordered, although the sequence seems to be more or less chronological. It would be helpful to know whether there is anything more significant about the order in which the protocols are presented, because it would enable the reader to give some sort of ‘weighting’ to each. The comments sections are on the whole very useful, giving information on response rates and some important comparisons with other protocols, together with a reference for each protocol.

There are some inaccuracies and omissions. For example, the AC regimen listed for breast cancer is not the one that most practicing oncologists would recognize or frequently use. The single agent activities listed at the beginning of each chapter are useful but, unfortunately, some contain inaccuracies, e.g. to ascribe cisplatinum a single agent response rate of only 27% in ovarian cancer is incorrect. In the section on osteosarcomas in protocols using high-dose methotrexate, the folinic acid rescue regimens are unclear and, despite the fact this is covered in the introductory section, this is of such importance that I think it should be emphasized within each protocol using high-dose methotrexate. In the section on Ewing’s sarcoma, the now commonly used IVAD/IVA and EVAIA protocols are not included – which they should be for this rare tumour. There are some inaccuracies in the section on non-small-cell lung cancer, soft tissue sarcomas and osteosarcoma which could be a little more serious if attempts were made to implement them by practicing oncologists. Discrepancies between this book and the clinical papers it cites could lead patients to be over- or under-treated.

As a comprehensive reference book and short guide to the majority of chemotherapy protocols, this book works well enough and will find a place as a reference book in oncology pharmacies and oncology libraries, and on the shelves of practicing oncologists. However, despite the disclaimer by the authors and publishers at the start, the inaccuracies that it contains should be addressed.

*Helena Earl*
*Oncology Centre*
*University of Cambridge*
*Box 193 Addenbrooke's Hospital*
*Hills Road*
*Cambridge CB2 2QQ*

### Lung Cancer, Second Edition

Edited by JA Roth, JD Cox and WK Hong, Publication Details: Price £75.00, ISBN 0–86542–573–6. Blackwell Science: UK, 1998.

This book is the second edition of a book which aims to cover all aspects of lung cancer. It has been updated over the last 5 years and edited by authors from the MD Anderson Cancer Centre, Texas, USA.

This book would aim to pitch itself as an intermediate work of reference, i.e. it should be a source of information more detailed than a general oncology textbook but fall short of the most recent advances in lung cancer which by their very nature would have to be published in peer reviewed journals. There are 20 chapters that cover most areas of lung cancer. A large number of chapters focus on developing strategies in lung cancer such as molecular and fluorescent detection, chemoprevention, lung cancer genetics and growth factor and biologic approaches to therapy. The more standard treatment modalities for lung cancer, such as surgery, radiotherapy and chemotherapy, are covered but there are some notable omissions which will be discussed later.

The early chapters include the biology of pre-neoplastic regions, and give a very detailed and good historical background to the subject. In particular, there is a good summary of the chromosomal abnormalities present and some discussion on how multistage carcinogenesis may apply in this disease. The chapter

